# Encephalitis Surveillance through the Emerging Infections Program, 1997–2010

**DOI:** 10.3201/eid2109.150295

**Published:** 2015-09

**Authors:** Karen C. Bloch, Carol A. Glaser

**Affiliations:** Vanderbilt University, Nashville, Tennessee, USA (K.C. Bloch);; Kaiser Permanente, Oakland, California, USA (C.A. Glaser)

**Keywords:** EIP, Emerging Infections Program, encephalitis, surveillance, bacteria, viruses

## Abstract

Encephalitis is a devastating illness that commonly causes neurologic disability and has a case fatality rate >5% in the United States. An etiologic agent is identified in <50% of cases, making diagnosis challenging. The Centers for Disease Control and Prevention Emerging Infections Program (EIP) Encephalitis Project established syndromic surveillance for encephalitis in New York, California, and Tennessee, with the primary goal of increased identification of causative agents and secondary goals of improvements in treatment and outcome. The project represents the largest cohort of patients with encephalitis studied to date and has influenced case definition and diagnostic evaluation of this condition. Results of this project have provided insight into well-established causal pathogens and identified newer causes of infectious and autoimmune encephalitis. The recognition of a possible relationship between enterovirus D68 and acute flaccid paralysis with myelitis underscores the need for ongoing vigilance for emerging causes of neurologic disease.

## Abstract 2

A 3-state syndromic surveillance system documented reduction and emergence of neurologic infectious diseases.

Encephalitis is a potentially devastating illness: the related case-fatality rate in the United States is >5% (*1*), and substantial neurologic disability is common among survivors. Historically, this syndromic illness has been difficult to diagnose: an etiologic agent was identified in <50% of encephalitis cases in the United States diagnosed during 1987–1998 (*2*). A major barrier to diagnosis during that period was the lack of sensitive, noninvasive laboratory techniques to identify central nervous system pathogens. However, by the early 1990s, PCR was proven to be comparable to brain biopsy for the diagnosis of herpes simplex virus (HSV) encephalitis, without the need for an invasive surgical procedure (*3*). There was optimism that application of PCR could also improve microbiologic diagnoses of encephalitis infections caused by other pathogens and, by extension, the outcome of the illnesses. The Emerging Infections Program (EIP), which is funded by the Centers for Disease Control and Prevention, initiated the Encephalitis Project, a syndromic surveillance program for encephalitis in existing EIP sites beginning in New York in 1997, California in 1998, and Tennessee in 2000, and all ending by 2010.

## Materials and Methods

Researchers from the 3 sites collaborated to develop shared inclusion criteria that captured both infectious and post-infectious syndromes such as acute disseminated encephalomyelitis (ADEM), using a case definition adapted from previous studies (Table 1). The case definition was constructed to maximize sensitivity, acknowledging that a proportion of cases meeting the EIP standardized definition may have had other conditions known to mimic encephalitis. Common exclusion criteria included patient age <6 months, immunocompromised status (HIV/AIDS or transplantation), and outpatient status. The California and Tennessee EIP sites collected comparable demographic, epidemiologic, and clinical information that was able to be aggregated for combined data analysis (*5*). The New York site focused on development of molecular diagnostic assays (*6**–**8*).

**Table 1:** Case definition for encephalitis in the Emerging Infections Program Encephalitis Project, 1997–2010*

Criteria
Major criterion (required): Altered mental status lasting ≥24 h
Plus >1 of 6 minor criteria:
1. Fever ≥38°C occurring ≤72 h before or after hospital admission
2. Seizures
3. Focal neurologic deficits not previously present on examination
4. Cerebrospinal fluid pleocytosis (≥5 leukocytes/mm^3^)
5. Abnormal electroencephalogram
6. Abnormal neuroimaging (computed tomography or magnetic resonance imaging) representing an acute process

*International Encephalitis Consortium case definition requires the presence of the major criteria plus ≥3 minor criteria for confirmed/probable; ≥2 for probable encephalitis (*4*).

A major goal of the EIP Encephalitis Project was the implementation of a standardized diagnostic algorithm to be used at all 3 sites. However, early in the course of the project, it was recognized that there were substantial regional differences in the frequency of specific pathogens, such as arboviral and rickettsial infections. The concept of a standardized testing algorithm thus evolved to include a site-specific core set of routinely performed laboratory tests to capture the most common and most treatable etiologies, supplemented by targeted testing on the basis of season, exposures, clinical features, and geography (Table 2) (*9*). For instance, Powassan virus, a tickborne pathogen endemic to the northern United States and Canada and therefore not included in the core algorithm of the Tennessee Unexplained Encephalitis Project, was diagnosed in a patient from New York who became ill during a trip to Tennessee, underscoring the importance of a thorough travel history to guide laboratory evaluation (*10*). This concept of tiered or individualized testing has subsequently been endorsed in management guidelines by the Infectious Diseases Society of America (*11*) and in a consensus statement of the International Encephalitis Consortium, an ad hoc group of subject matter experts and patient representatives (*4*).

**Table 2:** Core diagnostic testing algorithm for the Emerging Infections Program Encephalitis Project, 1997–2010*

**Pathogen**	**Specimen type**	**Test type**	**Seasonality**
**Viruses**			
** Adenovirus**	NP swab	PCR	Year-round
** Arbovirus panel†**	Serum	Serology	May–October
** Enteroviruses**	CSF	PCR	Year-round
	NP swab	PCR	Year-round
	Rectal swab	PCR	Year-round
** Epstein-Barr virus**	CSF	PCR	Year-round
	Serum	Serology	Year-round
** Herpes simplex virus 1 and 2**	CSF	PCR	Year-round
** Human herpesvirus 6**	CSF	PCR	Year-round
** Influenza virus A and B **	NP swab	PCR	November–April
** Parainfluenza virus 1–3 **	NP swab	PCR	November–April
** Rotavirus**	Rectal swab	Antigen	November–April
** Varicella zoster virus**	CSF	PCR	Year-round
** West Nile virus**	CSF	Serology	May–October
	Serum	Serology	May–October
**Bacteria**			
***Bartonella* spp.**	Serum	Serology	Year-round
*** Chlamydia pneumoniae***	NP swab	PCR	Year-round
***Ehrlichia* spp.**	Whole blood	PCR	May–October
	Serum	Serology	May–October
*** Mycoplasma pneumoniae***	NP swab	PCR	Year-round
	Serum	Serology	Year-round
** Rickettsia spp.**	Serum	Serology	May–October
*** Treponema pallidum***	CSF	VDRL	Year-round
	Serum	RPR	Year-round

*Diagnostic testing algorithm at the Tennessee site; regional differences and testing availability associated with minor variations in core testing at the California site. Additional supplementary testing was performed when indicated based on individualized epidemiologic, demographic, clinical, or radiographic features. CSF, cerebrospinal fluid; NP, nasopharyngeal; VDRL, venereal disease research laboratory test; RPR, rapid plasma reagin. **†Arbovirus panel included Lacrosse virus, St. Louis encephalitis virus, Western equine encephalitis virus, and Eastern equine encephalitis virus.**

A final unique feature of the EIP Encephalitis Project was the development of defined a priori pathogen-specific criteria to establish causality. Cases were classified as having a possible, probable, or confirmed etiology constructed on the basis of whether the identified pathogen was a well-established cause of encephalitis and whether there was direct evidence of central nervous system infection (*12*). For example, mycoplasma infection was the single most common infectious etiology identified in the California Encephalitis Project; however, in most cases, the diagnosis was based on serologic test results with no corroborating evidence of neuroinvasive disease; therefore, these cases were classified as having a “possible” diagnosis (*13*).

### Encephalitis Profiles

Although the findings for all patients enrolled in the study met the encephalitis case definition, there was tremendous heterogeneity in the clinical characteristics and outcomes of the cases. The large numbers of patients in these projects facilitated recognition of discrete clinical patterns. For example, temporal lobe abnormalities were predictive of HSV encephalitis. It was hypothesized that similar patterns might represent clinical clues to other infectious causes; ultimately, several subsets that had particular characteristics, referred to as encephalitis profiles, were identified (Table 3) (*14*). Although none of these profiles were found to be pathognomonic for a single pathogen, this schema has yielded new insights into the epidemiology and potentially to the treatment of subsets of patients who have encephalitis. For instance, the California Encephalitis Project identified a group of patients with profound refractory seizures, accounting for 5% of all cases enrolled at this site (*15*). This profile, subsequently characterized as idiopathic catastrophic epileptic encephalopathy or febrile infection-related epilepsy syndrome, is now widely acknowledged as a particularly severe form of encephalitis. Although the cause of this syndrome remains unknown, by identifying this unique phenotype, promising therapies such as initiation of a ketogenic diet have been identified (*16*).

**Table 3:** Emerging Infections Program Encephalitis Project clinical profiles, 1997–2010*

**Clinical profile**	**Patient description**	**Comments**
**Focal**		
** Temporal lobe**	Temporal lobe enhancement on imaging or activity on EEG	HSV accounted for approximately one third of cases
** Extrapyramidal**	Movement disorder	Measles (SSPE), autoimmune encephalitides
** Cerebellar**	Ataxia or gait disorder, or focal cerebellar lesion on imaging	Acute EBV infection seen in a minority of cases
**Generalized**		
** Cerebral edema**	Neuroimaging showing diffuse brain edema	Deaths exceed 70%
** Intractable seizures**	Seizures requiring anesthetic coma for management	Majority of case-patients: pediatric patients with prolonged hospitalization
** Seizure with rapid recovery**	Onset with seizure and return to baseline mental status in <96 h	CSF typically bland; *Bartonella* spp. most common cause (cat-scratch encephalopathy)
** Psychosis**	New onset of prominent psychiatric symptoms	Anti-NMDAR antibodies common in this syndrome

*EEG, electroencephalogram; HSV, herpes simplex virus; SSPE, subacute sclerosing panencephalitis; EBV, Epstein-Barr virus; NMDAR, anti-N-methyl-D-asparate receptor.

### Unexplained Cases

The EIP Encephalitis Project represents the largest cohort of patients (>5,000) with encephalitis studied to date: >4,000 case-patients were enrolled in the California Encephalitis Project and >700 in the Tennessee Unexplained Encephalitis Project. (Cases at the New York site were enrolled for diagnostic testing only.) Despite the rigorous diagnostic testing algorithm, in approximately half of all cases, no underlying cause for encephalitis was identified. Several factors likely contribute to the frustratingly high proportion of cases that had unidentified pathogens. Foremost is that, for many pathogens other than HSV, PCR of cerebrospinal fluid (CSF) was not an optimal diagnostic test. The high sensitivity of PCR in some instances lead to detection of reactivated viruses in CSF of questionable significance, such as Epstein-Barr virus (*17*) and human herpesvirus 6 (*18*). Also, for many organisms, serologic testing was superior to PCR, but antibodies were often not detectable until several weeks after the acute infection, and serum samples from the convalescent period was not always available. Issues related to specimen integrity such as volume, storage, and timing of collection likely contributed to inability to identify a cause in some cases. Finally, it has become increasingly clear that >5% of case-patients in whom encephalitis was presumed to have been caused by an infectious organism actually had autoimmune encephalitis. Retrospective testing of specimens from case-patients with undiagnosed disease in the California Encephalitis Project identified a newly described autoimmune syndrome, termed anti–N-methyl-D-asparate receptor (NMDAR) encephalitis, as the leading cause of encephalitis among patients <30 years of age (*19*). Our initial supposition that the large proportion of undiagnosed cases might be caused by the presence of undiscovered pathogens does not appear to be the case; independent testing of numerous samples at several research laboratories using multiple different techniques, including next-generation sequencing, did not identify any novel infectious agents.

## Results

### Established Causes of Encephalitis

A confirmed or probable cause of encephalitis was identified in approximately one third of cases studied. HSV, the most frequent cause of sporadic encephalitis in the United States (*1*), was underrepresented in this cohort, reflecting a referral bias toward more diagnostically challenging cases. In fact, clinician referral to one of the EIP encephalitis projects was often deferred until a commercially available HSV PCR test returned negative results, which led to the recognition that HSV PCR analysis early in the disease course could represent a false-negative result (*20*). On the basis of this observation, the recommendation for repeat HSV PCR on a subsequent CSF specimen for patients whose symptoms indicate a high clinical suspicion for HSV encephalitis has been incorporated into national management guidelines (*11*).

The substantial number of patients with encephalitis identified through this project enabled robust pathogen-specific case series of well-established but relatively rare causes of encephalitis. These included large series of patients with enteroviral encephalitis (*21*), tuberculosis meningoencephalitis (*22*), and amebic granulomatous encephalitis (*23*). Furthermore, the project was able to explore the putative association of several organisms for which a causal relationship with encephalitis remains tenuous, such as rotavirus (*24*), human metapneumovirus (*25*), and *Mycoplasma pneumoniae* (*13*). Although the latter organism remains a controversial cause of encephalitis because of the difficulty in demonstrating neuroinvasion, the frequency with which it is detected, particularly in children, has led to the inclusion of *Mycoplasma* serologic testing as part of the recommended pediatric testing algorithm (*4*).

### Autoimmune Cases of Encephalitis

Before the start of the EIP Encephalitis Project, it was well recognized that paraneoplastic syndromes could cause limbic encephalitis, albeit infrequently. In 2007, Josep Dalmau and colleagues described anti-NMDAR encephalitis, a novel form of autoimmune encephalitis (*26*). This syndrome was initially reported in women with ovarian teratomas and was believed to represent a paraneoplastic phenomenon. Testing of residual samples from the California Encephalitis Project confirmed that anti-NMDAR encephalitis affects a much broader spectrum of patients, including male and pediatric patients without a neoplastic antigenic stimulus (*27*). Among patients <30 years of age, anti-NMDAR encephalitis accounted for more cases than HSV, West Nile virus (WNV), and varicella zoster virus combined (*19*). A recent study showed that HSV encephalitis may serve as an antigenic trigger for subsequent development of anti-NMDAR encephalitis (*28*).

### Vaccine-Preventable Cases of Encephalitis

The widespread implementation of the varicella vaccine in the 1990s has essentially eliminated varicella zoster virus as a cause of pediatric encephalitis (*29*). Although various immunizations have been linked to encephalitis (*30*), a large review of pediatric cases showed no temporal relationship between vaccination and subsequent encephalitis, confirming the rarity of vaccine-associated neurologic complications (*31*). This finding, coupled with the identification of encephalitis as a potentially fatal complication of vaccine-preventable infections such as measles (*32*) and influenza virus (*33*), highlights the critical importance of universal immunization.

### Emerging Pathogens and Syndromes

When the EIP Encephalitis Project was initiated, it was unforeseeable that a virus never before identified in the Western Hemisphere would cause an encephalitis epidemic in the United States. Yet, during 1999–2013, more than 17,000 cases of West Nile neuroinvasive disease were diagnosed; the case-fatality rate was 9% (*34*). After the emergence of WNV in 1999, the New York site was uniquely positioned to assist with the identification of this unexpected pathogen, and to perform surveillance for additional cases (*35*). As the virus spread throughout the continental United States, the large numbers of patients referred to the California Encephalitis Project enabled analysis of WNV encephalitis among pediatric patients (*36*) and identification of risk factors predisposing to neuroinvasive disease (*37*).

The infrastructure that proved invaluable in enabling a rapid response to the WNV epidemic also was instrumental in identifying an emerging neurologic syndrome characterized by acute flaccid paralysis. In 2012, several physicians familiar with the California Encephalitis Project contacted the project, reporting cases of previously healthy patients with acute onset of a polio-like illness. Routine testing for organisms associated with acute flaccid paralysis returned negative results, raising concern for a novel agent or pathogen causing this syndrome. These sporadic cases occurred at geographically disparate sites and likely would not have been recognized without an existing surveillance mechanism. Ultimately, more than 23 cases were identified in California (*38*). The sentinel cluster of cases in California triggered national surveillance, resulting in 88 cases identified to date in 32 states (*39*). Investigation is ongoing, and although no causative pathogen has been identified, enterovirus D68 has been implicated in several cases (*40*).

## Discussion

The EIP Encephalitis Project has demonstrated the value of syndromic surveillance in a constantly changing environment. Globally, this project represents the largest known cohort of patients with encephalitis. The robust sample size provided sufficient power to investigate recognized pathogens and to identify newer causes of encephalitis, both infectious and autoimmune. Syndromic surveillance confirmed that previously common causes of pediatric encephalitis such as VZV have been all but eliminated by vaccination, and conversely, that childhood immunization is not substantially associated with development of encephalitis. The recognition of an emerging syndrome of acute flaccid paralysis with myelitis, possibly caused by enterovirus D68, underscores the need for ongoing vigilance for emerging causes of neurologic disease.
